# Interleukin-7 deficiency in rheumatoid arthritis: consequences for therapy-induced lymphopenia

**DOI:** 10.1186/ar1452

**Published:** 2004-11-16

**Authors:** Frederique Ponchel, Robert J Verburg, Sarah J Bingham, Andrew K Brown, John Moore, Andrew Protheroe, Kath Short, Catherine A Lawson, Ann W Morgan, Mark Quinn, Maya Buch, Sarah L Field, Sarah L Maltby, Aurelie Masurel, Susan H Douglas, Liz Straszynski, Ursula Fearon, Douglas J Veale, Poulam Patel, Dennis McGonagle, John Snowden, Alexander F Markham, David Ma, Jacob M van Laar, Helen A Papadaki, Paul Emery, John D Isaacs

**Affiliations:** 1 Molecular Medicine Unit, University of Leeds, Leeds, UK; 2 Academic Unit of Musculoskeletal Disease, Leeds General Infirmary, Leeds, UK; 3 Department of Rheumatology, Leiden University Medical Center, Leiden, The Netherlands; 4 Hematology Department, St Vincent Hospital, Sydney, Australia; 5 Cancer Research UK, University of Leeds, Leeds, UK; 6 Department of Haematology, Royal Hallamshire Hospital, Sheffield, UK; 7 Department of Hematology, University of Crete School of Medicine, Heraklion, Crete, Greece; 8 School of Clinical Medical Sciences (Musculoskeletal Research Group), The University of Newcastle, Newcastle upon Tyne, UK

**Keywords:** immune reconstitution, interleukin-7, T-cell differentiation, therapeutic lymphodepletion

## Abstract

We previously demonstrated prolonged, profound CD4^+ ^T-lymphopenia in rheumatoid arthritis (RA) patients following lymphocyte-depleting therapy. Poor reconstitution could result either from reduced *de novo *T-cell production through the thymus or from poor peripheral expansion of residual T-cells. Interleukin-7 (IL-7) is known to stimulate the thymus to produce new T-cells and to allow circulating mature T-cells to expand, thereby playing a critical role in T-cell homeostasis. In the present study we demonstrated reduced levels of circulating IL-7 in a cross-section of RA patients. IL-7 production by bone marrow stromal cell cultures was also compromised in RA. To investigate whether such an IL-7 deficiency could account for the prolonged lymphopenia observed in RA following therapeutic lymphodepletion, we compared RA patients and patients with solid cancers treated with high-dose chemotherapy and autologous progenitor cell rescue. Chemotherapy rendered all patients similarly lymphopenic, but this was sustained in RA patients at 12 months, as compared with the reconstitution that occurred in cancer patients by 3–4 months. Both cohorts produced naïve T-cells containing T-cell receptor excision circles. The main distinguishing feature between the groups was a failure to expand peripheral T-cells in RA, particularly memory cells during the first 3 months after treatment. Most importantly, there was no increase in serum IL-7 levels in RA, as compared with a fourfold rise in non-RA control individuals at the time of lymphopenia. Our data therefore suggest that RA patients are relatively IL-7 deficient and that this deficiency is likely to be an important contributing factor to poor early T-cell reconstitution in RA following therapeutic lymphodepletion. Furthermore, in RA patients with stable, well controlled disease, IL-7 levels were positively correlated with the T-cell receptor excision circle content of CD4^+ ^T-cells, demonstrating a direct effect of IL-7 on thymic activity in this cohort.

## Introduction

Peripheral blood T-cell lymphopenia is long-lasting in patients with rheumatoid arthritis (RA) receiving lymphodepleting therapies, such as monoclonal antibodies [1-3] or high-dose cyclophosphamide with autologous stem cell rescue (autologous stem cell transplantation) [4,5]. It has now been extensively documented in a number of systems that IL-7 drives the survival and proliferation of human T-cells after lymphodepletion (for review [6]). In particular, high circulating levels of this cytokine have been documented in patients rendered lymphopenic either by lymphocytotoxic treatment [7] or by HIV infection [8-10]. IL-7 produced in response to lymphopenia stimulates proliferation of both naïve and memory human T-cells [7], but also has a direct stimulating effect on thymic activity [11]. IL-7 plays many other roles such as the induction/enhancement of a T-helper-1 immune response [12,13], maturation of monocytes into dendritic cells, recruitment and expansion of T-cell clones [14-16], and induction of natural killer cell lytic activity [17-19]. These make IL-7 a master modulator of T-cell-mediated immune responses, particularly in tumour surveillance and eradication, in addition to its role as master regulator of peripheral T-cell homeostasis [8]

Specific abnormalities within the naïve T-cell compartment in RA, such as repertoire contraction and shortened telomeres, have suggested a possible defect in generating and/or maintaining naive T-cells [20-23]. Furthermore, we recently showed [24] that RA patients possessed fewer naïve CD4^+ ^T-cells than did healthy control individuals and that a smaller proportion of these cells contained a T-cell receptor excision circle (TREC). Circulating C-reactive protein (CRP) levels correlated inversely with the TREC content of naïve CD4^+ ^T-cells, suggesting that inflammation was driving naïve CD4^+ ^T-cell proliferation and differentiation, leading to dilution of TREC-containing cells. We could not, however, exclude an additional intrinsic defect in thymic T-cell production in RA patients [24].

In recent studies we reported persistent and profound CD4^+ ^T-cell lymphopenia in RA patients as long as 7 years after a single course of CAMPATH-1H monoclonal antibody treatment [25] and up to 36 months after autologous stem cell transplantation [26]. RA patients usually reconstitute their B and natural killer cells rapidly, whereas CD8^+ ^T-cell reconstitution takes longer and full recovery of CD4^+ ^T cells may never occur. This is in contrast to patients undergoing bone marrow or stem cell transplantation for haematological malignancy or solid tumours, in whom both T-cell compartments reconstitute within 1 year of follow up [27-29]. Poor reconstitution after lymphodepleting therapy is likely to result either from reduced *de novo *T-cell production from the thymus or from poor peripheral expansion of naïve and memory cells, both of which processes are driven by IL-7.

Here we report on a deficit in circulating levels of IL-7 in a cross-section of RA patients. This is associated with a reduced production of IL-7 in bone marrow derived stromal cell cultures, and may contribute to the defective CD4^+ ^T-cell reconstitution that occurs following therapeutic lymphodepletion, primarily at the level of mature T-cell expansion in the periphery. Furthermore, we show that TREC levels correlate with circulating levels of IL-7 in patients in whom inflammation is controlled.

## Methods

### Patient cohorts

Ethical approval for the project was obtained from the Leeds Teaching Hospitals National Health Service Trust Ethics Committee, and informed consent was obtained from each participant. Healthy control individuals were recruited from among local blood donors (*n *= 34). RA (*n *= 28) and osteoarthritis (OA; *n *= 12) patients were recruited through routine clinics at the Leeds General Infirmary (Table 1). They included patients with early, drug naïve (*n *= 7) and long-lasting, refractory (*n *= 21) RA (CRP range 5–155 mg/l) and patients with established, long-lasting OA (*n *= 12; CRP below detection range).

For the reconstitution studies we analyzed three RA patient cohorts (*n *= 31) and a cohort of non-RA patients with solid tumours (*n *= 7; Table 2). Each RA patient received high-dose cytotoxic therapy followed by autologous haematological transplants [26,30,31]. Each had disease that had proved resistant to multiple conventional antirheumatic drugs. Cohort 1 received an unmanipulated graft; cohort 2 received a graft that had undergone selection for CD34^+ ^cells; and cohort 3 received a graft that had been CD34^+ ^cell selected and T-cell depleted. The clinical progress of these patients was previously described elsewhere [26,30,31]. Control patients (Table 2) included five individuals with lung carcinoma, one with breast carcinoma and one with melanoma. They received unmanipulated autologous grafts following high-dose chemotherapy, as previously documented [32]. For the IL-7 longitudinal studies, we analyzed four lymphoma and three sarcoma patients. All received intensive chemotherapy followed by reinfusion of unmanipulated autologous stem cells (Table 2). In addition, we studied three patients with systemic vasculitis who received the lymphocytotoxic monoclonal antibody CAMPATH 1H [33].

For our work on RA patients in clinical remission (Table 1), we recruited consecutive patients (*n *= 36) attending the rheumatology outpatient clinics with stable RA. They possessed no clinically significant synovitis and were deemed to be in 'remission' by the assessing consultant rheumatologist. Patients satisfied all of the following inclusion criteria: previous certified diagnosis of RA; over 18 years of age; disease duration of at least 12 months before remission; no disease flare within preceding 6 months; stable treatment within preceding 6 months; nil or minimal clinical evidence of active inflammatory disease and CRP below 15 mg/l within preceding 6 months; and no clinical indication to change treatment. We further refined this cohort by separating patients into those who satisfied the American College of Rheumatology (ACR) remission criteria and those who did not (Table 3).

### Cytokine measurements

IL-7, transforming growth factor-β_1_, IL-6, tumour necrosis factor (TNF)-α and oncostatin M levels in sera and in tissue culture supernatants were measured using enzyme-linked immunosorbent assay (ELISA; R&D, Abingdon, UK), in accordance with the manufacturer's instructions. The sensitivities of the assay were <0.1 pg/ml for IL-7, 0.2 pg/ml for IL-6, 0.5 pg/ml for TNF-α, and 20 pg/ml for oncostatin M.

### T-cell subset separation

Peripheral blood mononuclear cells (PBMCs) were recovered as described previously [24], and CD4^+ ^and CD8^+ ^T cells were separated by negative selection (Metachem, Meylan, France). Purified CD4^+ ^and CD8^+ ^T cells (>92% pure for CD4^+ ^and 89% pure for CD8^+ ^T cells) were stained for CD45RB (FITC; Dako, Ely, UK), CD45RA (PE; Serotec, Oxford, UK), CD45RO (PE-CY5; Serotec) and CD62L (ECD Coulter, High Wycombe, UK) using conventional methods. Naïve T-cells were further sorted according to their CD45RB^bright^, CD45RA^+ ^and CD62L^+ ^phenotype, using a FACS-Vantage cell sorter (Becton Dickinson, Oxford, UK). Memory cells and other subsets were identified based on their expression of CD45RB^bright/dull^, CD45RA^±^, CD45RO^bright/dull^, and CD62L^± ^[24].

### Real-time polymerase chain reaction quantification of T-cell receptor excision circles

DNA was extracted from the different lymphocyte populations using standard proteinase K digestion followed by a phenol/chloroform extraction, either from total CD4^+ ^and CD8^+ ^populations after magnetic separation or from naïve cells after further cell sorting. TRECs were quantified using a real-time polymerase chain reaction based assay, as described previously [24]. Briefly, TREC primers were F (d-CAC CTC TGG GCT ACG TGC TAG) and R (d-GAA CAC ATG CTG AGG TTT AAA GAG AAT); and glyceraldehyde-3-phosphate dehydrogenase primers were F (D-AAC AGC GAC ACC CAT CCT C) and R (d-CAT ACC AGG AAA TGA GCT TGA CAA). This analysis provided a final value that represented TREC DNA as a proportion of glyceraldehyde-3-phosphate dehydrogenase DNA, which is equivalent to the percentage of cells containing a TREC. Following the release of the entire T-cell receptor locus sequence late in 2002, we validated our assay utilizing an alternative set of TREC primers designed to minimize background signal when using PBMC DNA.

### Proliferation assays

PBMCs were separated as above from 5 ml blood from RA patients and healthy control individuals. An aliquot of PBMCs was stained with a combination of CD127 (FITC; Serotec), CD19 (PE; Serotec) and CD4 or CD8 (PE-CY5; Serotec) to quantify IL-7 receptor expression on different cell types by flow cytometry. Cells were resuspended in RPMI 1640 supplemented with penicillin and streptomycin, glutamine and 10% human AB^+ ^serum (Sigma, Aldwich, UK) and proliferation was assessed in response to PHA (10 μg/ml, Sigma), IL-2 (20 units/ml; Sigma), IL-7 (1–100 ng/ml; Sigma) or anti-CD3 antibody (OKT3; 1 μg/ml) with or without anti-CD28 antibody (YTH913.12; 5 μg/ml) co-coated on plastic Proliferation was quantified by incorporation of ^3^H-thymidine (1 μCi/well) after 5 days of culture.

### Long-term bone marrow cultures

Bone marrow mononuclear cells were obtained from posterior iliac crest aspirates from RA patients and healthy control individuals after informed consent had been obtained (with local research ethics committee approval), following centrifugation on Lymphoprep (Nycomed Pharma AS, Oslo, Norway), as previously described [34,35]. Aspirates from RA patients were repeated after 6–8 months of therapy with infliximab (Remicade; Schering Plough, Kenilworth, NJ, USA). Long-term bone marrow cultures from 10^7 ^bone marrow mononuclear cells were grown, in accordance with standard techniques [34,35]. By allowing the formation of an adherent layer consisting mainly of macrophages and cells of mesenchymal origin, this culture system has been considered appropriate for evaluating the regulatory role played by the bone marrow microenvironment in haematopoiesis [36]. At weekly intervals, cultures were fed by demi-depopulation. The adherent layer was usually confluent after 3–4 weeks, and at that time point cell-free supernatants were harvested and stored at -70°C for cytokine quantification.

### Statistical methods

Nonparametric tests were used throughout. The Mann–Whitney U-test for two independent samples was used to compare healthy control individuals with RA patients. The Spearman rank correlation coefficient was used to determine correlations between two variables. A Wilcoxon sign rank test was used to compare pretherapy and post-therapy outcomes.

## Results

### Basal interleukin-7 production is reduced in rheumatoid arthritis

**Figure 1:**
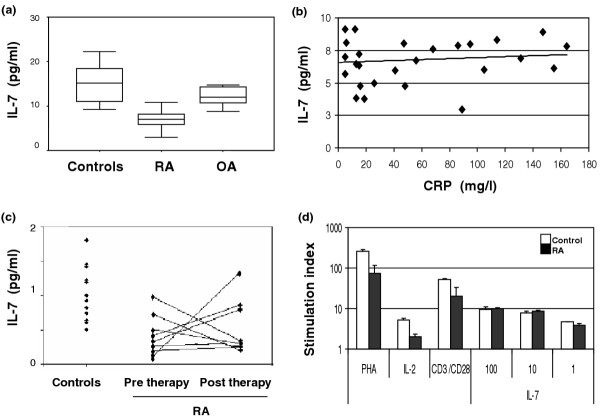
IL-7 deficiency in rheumatoid arthritis (RA). **(a) **IL-7 levels were measured in serum from 34 healthy control individuals (median age 46 years), 28 patients with RA (seven with recent onset RA before institution of therapy and 21 with established, refractory RA; median age 55 years) and 12 patients with established osteoarthritis (OA; median age 56 years). Control individuals had significantly higher levels of circulating IL-7 than did RA patients (*P *< 0.00001). OA patients tended to have lower IL-7 levels than healthy control individuals (*P *= 0.035) but higher than RA patients (*P *< 0.00001). **(b) **IL-7 levels were plotted against C-reactive protein (CRP) values for 28 patients with active RA, but no relationship could be identified (R = 0.201, *P *= 0.161). **(c) **Bone marrow was obtained by aspiration from the iliac crest from healthy control individuals (*n *= 15) and from RA patients (*n *= 8) before and after therapeutic tumour necrosis factor (TNF)-α blockade. Long-term bone marrow stromal cell cultures were established, and spontaneous IL-7 release was measured. Control bone marrow stromal cells released significantly more IL-7 than did RA marrow (*P *= 0.001). There was no consistent effect of anti-TNF-α therapy on IL-7 expression (paired pre-post treatment test). **(d) **Peripheral blood mononuclear cells from healthy control individuals (*n *= 3) and RA patients (*n *= 3) were cultured in the presence of PHA (10 μg/ml), IL2 (20 U/ml), anti-CD3 (1 μg/ml) plus anti-CD28 (5 μg/ml), or titrated doses of IL-7 (1–100 ng/ml), for 5 days. Proliferation was assayed by ^3^H-thymidine incorporation. RA and healthy cells responded similarly to IL-7, but RA cells were hyporesponsive to other stimuli.

We measured serum levels of IL-7 in a cross-section of active RA patients (*n *= 28), healthy control individuals (*n *= 34) and OA patients (*n *= 12). There was no correlation between serum levels of IL-7 and age in healthy control individuals [37,38], and sex did not make any difference. Circulating IL-7 levels (Fig. 1a) were significantly lower in RA patients than in healthy control individuals (*P *< 0.00001). In RA there was no association between levels of circulating IL-7 and disease duration, inflammation as measured by CRP (Fig. 1b; nonsignificant correlation [R = 0.201, *P *= 0.161]), presence of a shared epitope (*n *= 17), or antirheumatic therapy (nonsteroidal anti-inflammatory drugs, methotrexate, or steroids). OA patients exhibited slightly lower IL-7 levels than did control individuals (*P *= 0.035) but they had significantly higher IL-7 levels than did RA patients (*P *< 0.00001). After Bonferroni correction there was no longer a significant difference between control individuals and OA patients, but other results remained unaffected. Regression analysis did not reveal any further trends.

There are several sources of IL-7 production, including stromal cells in the bone marrow, dendritic cells and epithelial cells in the thymus, skin and gut [6]. We compared the ability of bone marrow stromal cells, derived from RA patients (*n *= 9) and healthy control individuals (*n *= 15), to produce IL-7 spontaneously in long-term cultures (Fig. 1c). The production of IL-7 was significantly lower in RA patients than in control individuals (*P *= 0.001). Furthermore, production did not consistently change after clinical remission induced by therapeutic TNF-α blockade (*n *= 8; *P *= 0.725). We also examined the PBMC response to IL-7 in RA patients and healthy control individuals. Whereas RA PBMCs responded suboptimally to IL-2, mitogen (PHA) or antigen (anti-CD3/CD28), as previously documented [39], their response to IL-7 was similar to that in control individuals (Fig. 1d). Importantly, in a cross-sectional comparison of 10 RA patients and 10 healthy control individuals, we could not find a significant difference in the number of cells expressing the IL-7 receptor (CD127) or in its level of expression (data not shown). Altogether, these findings suggest a deficit in circulating levels of IL-7 in RA, possibly due to an inability to produce IL-7, at least in stromal cells of bone marrow origin.

### Defective T-cell expansion in rheumatoid arthritis

Patients receiving lymphocytotoxic therapy for conditions other than RA reconstitute more rapidly and completely than do RA patients. We previously studied three cohorts of RA patients who had received high-dose chemotherapy followed by stem cell reinfusion (Table 2). As previously reported [26,30,31], CD4^+ ^counts fell after treatment and subsequently remained low in all cohorts, with no significant differences due to graft manipulation (data not shown). In contrast, CD8^+ ^T-cell counts initially rose before rapidly returning to basal levels.

**Figure 2:**
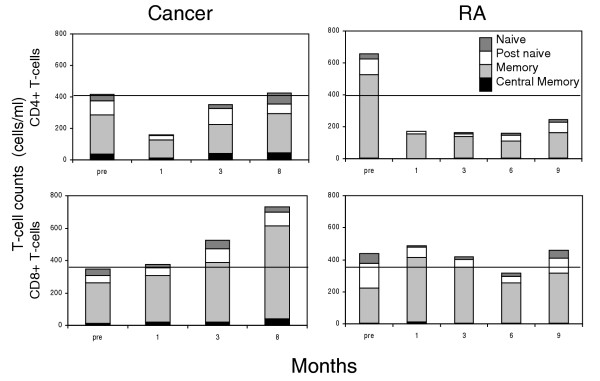
Poor T-cell expansion in rheumatoid arthritis (RA) patients. Phenotyping of isolated CD4^+ ^and CD8^+ ^T-cell populations was performed using the cell surface markers CD45RB, CD45RA, CD45RO and CD62L. Differentiation subsets were defined as naïve cells (grey bars: CD45RB^bright^, CD45RA^+^, CD45RO^-^, CD62L^+^), conventional memory cells and their precursors (striped bars: CD45RB^bright/dull^, CD45RA^-^, CD45RO^+^, CD62L^-^) and post-naïve intermediates (white bars: CD45RB^bright/dull^, CD45RA^-^, CD45RO^-/dull^, CD62L^+^), as described previously [24]. Presumed 'central' memory cells (black bars) are CD45RB^dull^, CD45RA^+^, CD45RO^+ ^and CD62L^+^. Total T-cell numbers are indicated by the height of the bars. Lines across the graphs indicate the lower limits of the normal range for CD4^+ ^and CD8^+ ^T-cell counts. Cancer patients (*n *= 7 solid tumours [Table 2]) reconstitute CD4^+ ^T cells largely by expansion of intermediate and memory subsets. This is not seen in RA patients (*n *= 12 at baseline and 1 month, *n *= 7 at 9 months; six patients from each of cohorts 2 and 3 [Table 2]). A similar expansion accounts for the 'overshoot' above baseline in CD8^+ ^T-cells in cancer patients, whereas only a minimal transient expansion of memory CD8^+ ^T-cells is observed in RA.

In the present study we compared T-cell reconstitution in 12 RA patients (six from each of cohorts 2 and 3) and seven patients with solid tumours (Fig. 2 and Table 2). To avoid potential confounding effects of immunosuppressive drugs, RA patients were removed from the analysis if it subsequently became necessary to reinstitute antirheumatic therapies at times when disease activity resumed. The figure therefore represents 12 RA patients pretreatment and seven at 9 months.

The chemotherapy regimens differed between RA and non-RA patients, but the nadir lymphocyte counts were similar. Figure 2 illustrates the composition of the peripheral T-cell pool at baseline and at various times after treatment. The individual subsets were defined according to the lymphocyte differentiation pathway suggested by our previous work [24]. The most naïve cells are represented in grey at the top of each bar chart. These cells progress to conventional memory cells and their precursors (striped bars) via post-naïve cells (white bars). Presumed 'central' memory cells are presented in black. Notably, at baseline RA patients possessed no CD4^+ ^and CD8^+ ^central memory cells in peripheral blood, as reported previously [24]. After chemotherapy there was simultaneous accumulation of all subsets in cancer patients, resulting in rapid restitution of CD4^+ ^T-cell counts within 3 months. The same was true of the CD8^+ ^subsets except that there was an 'overshoot' of memory CD8^+ ^T-cells. In contrast, there was no early expansion of any T-cell subset in RA, although some long-term restoration of naïve CD4^+ ^subsets was observed. Naïve CD8^+ ^T-cells also accumulated slowly, and there was a brief expansion of CD8^+ ^memory cells. These marked differences between RA and cancer patients demonstrate that a limited early peripheral expansion after treatment may account, in part, for the lack of T-cell reconstitution in RA.

Although graft manipulation differed between cancer patients (un-manipulated) and RA (CD34 selected ± T-cell depletion) as mentioned above, we found that graft manipulation did not affect the rate of reconstitution in RA patients (data not shown). Other factors that differ between the RA and control group reflect the underlying disease. For example, RA patients may have been exposed to low-dose corticosteroid therapy as part of their prior treatment. It is not possible to exclude an effect of such a factor on our data.

### Delayed thymic activity in rheumatoid arthritis

**Figure 3:**
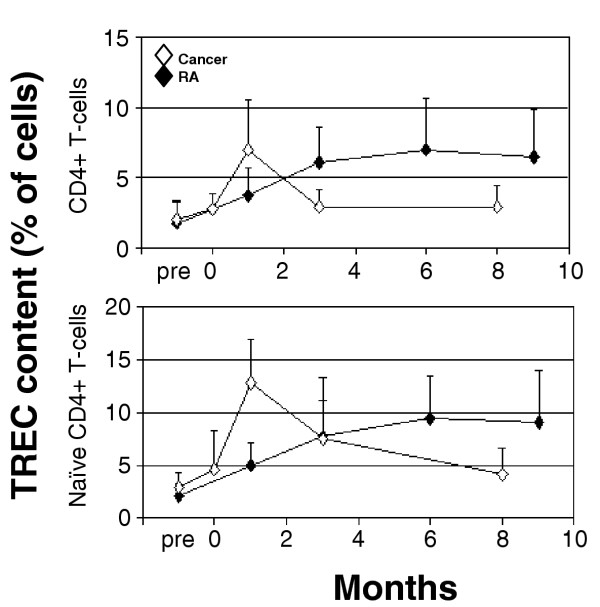
Thymic reserve in lymphopenic cancer and rheumatoid arthritis (RA) patients. The proportion of T cells containing a T-cell receptor excision circle (TREC) was measured longitudinally in cancer (*n *= 7 solid tumours [Table 2]) and RA (*n *= 12 at baseline and 1 month, six patients from both of cohorts 2 and 3 [Table 2]; and *n *= 7 at 9 months, three from cohort 2 and four from cohort 3) patients' pure CD4^+ ^T-cells and following cell sorting of naïve cells. In cancer patients TRECs rapidly rose within 1 month and then slowly returned to pretreatment levels by 8 months. In RA patients there was a slow but sustained rise in TRECs over that time, achieving similar peak levels to cancer patients by 9 months.

In order to compare thymic activity after lymphodepletion, we measured TRECs longitudinally in CD4^+ ^T-cells in the same RA and cancer patient cohorts. As a molecular marker of T-cell receptor rearrangement, TRECs provide a surrogate measure of recent thymic activity [40,41]. We measured TREC content in total CD4^+^-T cells as well as in naïve CD4^+^-T cells in isolation. Patterns of TREC variation were consistent between the seven cancer patients. TRECs rapidly accumulated after treatment but returned to baseline by 3 months (Fig. 3; open diamonds). Our data in cancer are therefore consistent with an early surge in thymic activity, followed by a slow return to baseline at a time when the T-cell counts have returned to baseline levels. Variation in the TREC content of naïve cells also followed that pattern. The reduction in TREC content of an individual subset such as naïve cells is better explained by proliferation within that subset [24,42,43], therefore suggesting that naïve T-cells underwent peripheral expansion, resulting in TREC dilution in CD4^+ ^cells (open diamonds). Similar results were observed for CD8^+ ^T cells (data not shown).

The early thymic response to lymphopenia did not occur in the 12 RA patients. In contrast, the TREC content of total CD4^+ ^T-cells climbed gradually for several months after treatment (Fig. 3; closed diamonds). The TREC measurements in naïve cells also did not return to baseline, however, suggesting a lack of proliferation of CD4^+ ^naïve cells. Therefore, a delay in achieving good release of newly developed T-cells also appeared to contribute to slow T-cell reconstitution in RA after high-dose chemotherapy. Similar results were observed for CD8^+ ^T-cells (data not shown).

### Lymphopenia-induced interleukin-7 production is defective in rheumatoid arthritis

**Figure 4:**
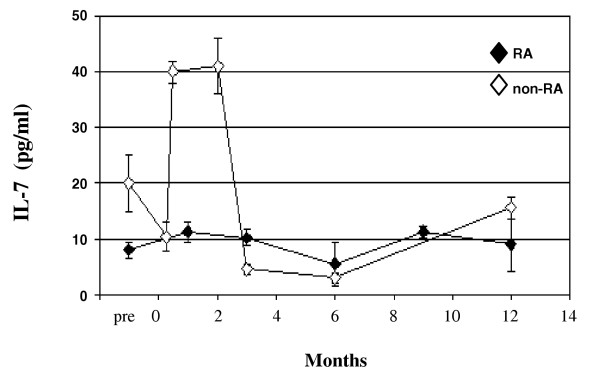
Lower circulating levels of IL-7 in rheumatoid arthritis (RA). IL-7 levels were measured in serum samples taken longitudinally from RA patients (*n *= 6 at baseline and 1 month, *n *= 4 subsequently; patients without relapse all from cohort 3) or patients with lymphoma, cancer or systemic vasculitis (*n *= 4 up to 3 month, *n *= 2 afterward), all of whom were lymphopenic for up to 3 months after lymphodepleting therapies. IL-7 circulating levels remained low in RA patients, compared with a substantial rise in the mixed cohort of non-RA patients.

Figures 2 and 3 suggest that the development and expansion of CD4^+ ^T-cells were compromised in lymphopenic RA patients. Both the development and expansion of T cells have been extensively documented in relation to IL-7 (for review [6]). The relative deficiency in circulating IL-7 levels in RA patients identified in Fig. 1 therefore suggests a significant role for IL-7 in impaired T-cell reconstitution following high-dose chemotherapy. We measured serum levels of IL-7 longitudinally in four RA patients after lymphodepleting therapy (cohort 3, without relapse within 12 months) and seven non-RA patients (Table 2). Figure 4 clearly demonstrates a four- to fivefold rise and subsequent decrease in IL-7 levels, coincident with short-term lymphopenia in non-RA patients (triangles). In marked contrast, IL-7 levels did not change significantly in four RA patients over 12 months of follow up (squares).

### Interleukin-7 levels correlated with thymic activity in patients with well controlled rheumatoid arthritis

In RA patients whose disease was controlled by *in vivo *TNF blockade, spontaneous release of IL-7 from bone marrow derived stromal cell cultures was variable, remaining reduced in some patients but returning to normal in others (Fig. 1). We therefore decided to investigate IL-7 levels in patients with well controlled disease and minimal levels of disease activity for at least 6 months before recruitment (*n *= 36; Table 1). Levels of IL-7 were heterogeneous and ranged from 2.47 to 16.25 pg/ml. No clinical parameter was significantly correlated with IL-7 levels (disease duration, remission duration, previous or current therapy, rheumatoid factor).

**Figure 5:**
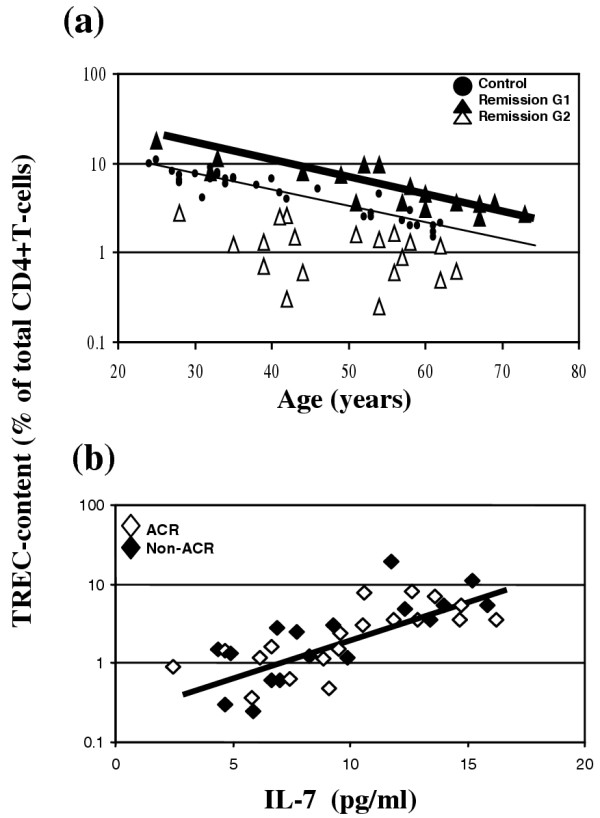
Circulating IL-7 levels are directly correlated with the TREC content of CD4^+ ^T-cells in rheumatoid arthritis (RA) patients in clinical remission. **(a) **The T-cell receptor excision circle (TREC) content of total CD4^+ ^T-cells, measured in patients in clinical remission (*n *= 36, all triangles [Table 1]), is heterogeneous, ranging from values observed in healthy control individuals to values in active RA patients. Using the age relationship to TREC content in healthy control individuals (black circles and thin line, correlation coefficient R = -0.816, *P *< 0.00001; previously reported [24]), two groups of patients can be differentiated: group 1 exhibits TREC content similar to or greater than that in age-matched healthy control individuals; and group 2 exhibits lower TREC content. We used the median value for TREC content to separate patients into two groups. We refer to these two groups as group 1 (G1; above median value, indicated by black triangles) and group 2 (G2; below median value, indicated by open triangles). The age relationship to TREC content is recovered only in group 1 (thick line; correlation coefficient R = -0.738, *P *= 0.001; for group 2 R = 0.341, *P *= 0.174). **(b) **Circulating IL-7 levels are directly correlated with TREC content of CD4^+ ^T-cells in 36 patients in clinical remission (R = 0.777, P < 0.00001). In addition, patients satisfying the American College of Rheumatology (ACR) criteria for remission are indicated by open diamonds and patients not satisfying the ACR criteria by closed diamonds (Table 3). These two groups are undistinguishable.

We measured TREC in total CD4^+ ^T-cells in these patients in clinical remission in relation to age. The results were also heterogeneous (Fig. 5a; all triangles). Comparing these values with our previous results in healthy control individuals (small circles [24]), there appeared to be two distinct patient groups. One of these groups had a CD4^+ ^T-cell TREC content similar to or higher than that in age-matched healthy control individuals, and the other group exhibited lower TREC content. We used the median TREC content to distinguish two groups. Open and closed triangles relate to group 1 (above the median TREC value) and group 2 (below the median TREC value), respectively. The relationship between TREC content and age was present in group 1 (thick line; R = -0.738, *P *= 0.001) but not in group 2. No clinical parameter was able to predict TREC content (disease duration, remission duration, previous or current therapy, rheumatoid factor).

We reanalyzed the IL-7 data with respect to this dichotomy in TREC levels, and there was a significant difference in circulating levels of IL-7 between these two groups (group 1, *n *= 17: IL-7 12.71 ± 2.76 pg/ml, range 9.57–16.25 pg/ml; group 2, *n *= 19: IL-7 6.50 ± 1.88 pg/ml, range 2.47–9.30 pg/ml; *P *< 0.00001). Furthermore, there was a positive correlation between the levels of circulating IL-7 and the TREC content of total CD4^+ ^T cells (Fig. 5b; *n *= 36, all diamonds; R = 0.777, *P *< 0.00001). No similar relationship was observed in healthy control individuals (*n *= 12; R = 0.219, *P *= 0.595).

We subsequently reanalyzed the data according to the ACR criteria for clinical remission [44,45]. Patients fulfilling or not fulfilling the ACR criteria (Table 3) are shown as open and black diamonds, respectively, in Fig. 5b. The two populations were undistinguishable in terms of TREC content (*P *= 0.807). There was no difference in their circulating levels of IL-7 (ACR positive: 9.07 ± 3.33 pg/ml, range 4.9–15.23 pg/ml; ACR negative: 9.31 ± 4.01 pg/ml, range 2.47–16.25 pg/ml; *P *= 0.838). Furthermore, the correlation between IL-7 and TREC content was maintained in both groups (ACR positive, *n *= 17: R = 0.680, *P *= 0.005; ACR negative, *n *= 19: R = 0.779, *P *= 0.001). These data suggest that, removing any influence of systemic inflammation, RA patients form two groups that are characterized by normal or low levels of thymic activity and IL-7. It is not possible to predict from these data whether these abnormalities are primary or, indeed, whether they have pathogenic significance. However, they may be important in the context of reconstitution capacity after lymphodepleting therapies.

## Discussion

We previously demonstrated that RA patients failed to reconstitute their peripheral T-cell pool even several years after lymphodepleting therapy [25,26,30,31]. The aim of the present work was to identify possible factors underlying this observation. IL-7 drives the expansion of human T-cells [8,46,47], and moreover it is an important thymic stimulant [11]. We identified a deficit in circulating levels of IL-7 in a cross-section of patients with active RA (Fig. 1). It was therefore possible that a similar deficit in IL-7 was a critical factor in the suboptimal response to lymphopenia in RA patients. Our data suggest that the RA thymus has a similar reserve to the thymus of disease control individuals (Fig. 3; similar peak levels at 9 months in RA as at 1 month in cancer), although it exhibits a more sluggish response to lymphopenia. However, both naïve and memory RA T-cells expand poorly in response to lymphopenia (Fig. 2), and this appears to be the major factor limiting reconstitution. We have also demonstrated low levels of lymphopenia-induced circulating IL-7 in RA patients (Fig. 4), and low basal IL-7 production from stromal cells originating from the bone marrow (Fig. 1). Finally, we showed a direct correlation between circulating levels of IL-7 and thymic capacity to produce new T-cells in RA patients with clinically undetectable disease activity (Fig. 5).

To date, IL-7 is not a cytokine that has been associated with RA. However, there are conflicting results regarding its expression in RA patients. In one study [48] IL-7 was present at high levels in the serum of adult RA patients, and it correlated with CRP. In contrast, in children with systemic juvenile RA, plasma levels of IL-7 were unrelated to disease activity (joint counts and circulating IL-6) and undetectable in synovial fluids [49]. In another study, IL-7 was elevated in RA synovial fluid but not in OA [50] and its production was associated with stromal cells in the synovium [51]. Circulating levels of IL-7 in healthy control individuals are also very heterogeneous between publications (ranging from 0.1 to 30 pg/ml), possibly because of the use of different ELISA systems (commercial IL-7 ELISA kits using monoclonal or polyclonal antibodies, in-house sandwich ELISA using polyclonal rabbit antisera). In our study we found that IL-7 levels were highly dependent on the condition of serum collection (in particular, the type of Vacutainer [Greiner Bio-one, Knemsmuster, Austria; standard NHS supply]) and we standardized our collection protocol (blood taken into plain glass tubes, clotting time of 2 hours at room temperature, centrifugation at 1000 *g *for 10 min, storage at -20°C). In addition, in a recent report from Fry and Mackall [8], circulating levels of IL-7 in CD4^+ ^T-cell depleted and repleted HIV patients were in keeping with our findings (<30 pg/ml and 10–20 pg/ml, respectively).

Peripheral T-cell expansion differed greatly between our patient groups, as shown in Fig. 2. This was particularly obvious for memory cells and their precursors, and appeared sufficient to account for the reconstitution defect in RA. However, lack of TREC dilution in naïve T-cells (Fig. 3) also suggested an absence of expansion within that subset in RA. IL-7 deficiency may again be relevant. IL-7 is produced in response to lymphopenia [7] and stimulates proliferation of both naïve and memory human T-cells. Although serum was not available from our cohort of solid tumour patients, we found high circulating levels of IL-7 in lymphodepleted patients with other tumours and with systemic vasculitis (Fig. 4), which is in keeping with the literature. In contrast, we found that basal serum IL-7 levels were reduced in a range of RA patients, irrespective of inflammation or medication (Fig. 1b). Furthermore, there was no IL-7 rise following lymphodepletion (Fig. 4). RA and control PBMCs responded equivalently to IL-7 stimulation *in vitro*, suggesting no defect in IL-7 receptor expression or signalling (Fig. 1d).

Circulating IL-7 levels may also reflect the availability of specific binding sites on T-cells [6], but our two patient groups were similarly lymphopenic, making this explanation unlikely. Lymph node-resident dendritic-like cells may also produce IL-7 [52]. Although we were unable to examine these cells directly, our data do not suggest compensatory production from that source. Therefore, although we cannot definitively exclude alternative explanations for reduced IL-7 levels, low levels in lymphopenic RA patients (Fig. 4) and the variable ability to recover IL-7 in remission (Fig. 5) strongly implicate an underlying defect in IL-7 regulation, also highlighted by the bone marrow derived stromal culture (Fig. 1). IL-7 expression is upregulated or downregulated by different cytokines in different tissues (transforming growth factor-β, interferon-γ, TNF-α, IL-1 and IL-2, among others) and further work is necessary to uncover the mechanisms that control circulating levels of IL-7.

CD8^+ ^lymphopenia is also associated with raised circulating IL-7 levels [53] but this correlation is less strong. This suggests that factors other than IL-7 can effectively drive CD8^+ ^T-cell expansion, and it is notable that transient expansion of CD8^+ ^memory T-cells did occur in RA patients. Our experience and that of others suggests that such expansions may be driven by intercurrent infections (Isaacs JD, unpublished observations) [54]. This may also underlie the CD8^+ ^T-cell over-compensation observed in cancer patients (Fig. 2).

The RA thymus was clearly capable of producing new T-cells. This was evident not only when comparing naïve T-cell reconstitution in RA and cancer cohorts (Fig. 2) but also when TREC-containing cells were examined (Fig. 3). There is a complex relationship between thymic activity, T-cell proliferation and death, and TREC measurements [24,42,43,55]. Just after lymphocytotoxic therapy, however, TREC levels and T-cell counts are low and their subsequent accumulation must therefore reflect thymic output. TRECs achieved similar peak levels in both RA and cancer patients, suggesting an equivalent thymic capacity for T-cell production in these two groups. In cancer patients, however, TREC levels peaked early, as compared with a slow rise in RA patients. An association between higher levels of IL-7 and thymic capacity to produce new T-cells was predictable, based on the direct stimulatory effect of IL-7 on thymic activity at many stages in T-cell progenitor development [6,11,56-60] High levels of IL-7, as detected in lymphopenic control patients, could therefore result in a burst of thymic activity. In contrast, it is not immediately obvious what other factor(s) could determine the delayed rise in thymic activity in RA patients. Other growth factors are also able to stimulate the thymus [61], but another plausible mechanism is the removal of inhibition. Several of the cytokines that are abundant in RA, such as IL-6, oncostatin M and leukaemia inhibitory factor, suppress thymic function [37]. Levels of TNF-α, IL-6 and oncostatin M fell after high-dose chemotherapy in RA patients (data not shown) as the disease entered remission, and this may have resulted in a corresponding slow increase in thymic activity.

Our data have pathogenic and therapeutic implications. First, they provide further support for a stromal cell function defect in RA. Previous studies of bone marrow progenitor cell reserve and stromal function in RA patients were more consistent with a defect secondary to TNF-α associated toxicity [34]. In those studies, progenitor cell reserve was reduced, and RA stroma was unable to support haematopoiesis from healthy CD34^+ ^progenitors. Both abnormalities correlated with TNF-α levels in bone marrow culture supernatants and significantly improved after *in vivo *TNF-α blockade. Those data therefore support a scenario in which the RA marrow was suppressed by chronic exposure to TNF-α and potentially other proinflammatory cytokines. However, our data relating both to circulating IL-7 and to bone marrow production demonstrate independence from the inflammatory process (Fig. 1) at least in some patients, and are consistent with a primary abnormality.

Therefore, supplementation with recombinant IL-7 may be necessary to improve lymphocyte reconstitution in lymphopenic RA patients, with the caveat that this cytokine is also a co-stimulatory factor for T-cells. It may therefore encourage the expansion of autoreactive T-cells with a worsening of disease. For example, IL-7 has been associated with preferential expansion [62] and activation [63] of autoreactive T-cells in multiple sclerosis. Additionally, IL-7 has been associated with lymphoproliferative disorders [64-66] to which RA patients are already predisposed. Furthermore, our data do not exclude additional contributions to limited T-cell expansion, and proliferative exhaustion is a factor that may not be amenable to therapeutic intervention. It is therefore possible that terminally differentiated memory T-cells, resulting from chronic immune activation in RA, cannot proliferate in response to lymphopenia. This does not explain the lack of proliferation of naïve T-cells from RA patients, however (Figs 2 and 3).

## Conclusion

In conclusion, although our data are necessarily an averaged view of events that occur after lymphodepletion, we have made a number of observations relevant to poor T-cell reconstitution in lymphopenic RA patients. Importantly, the RA thymus is capable of producing naïve T-cells but its function is compromised by an IL-7 deficiency. The latter also severely limits the peripheral expansion of both naïve and memory T-cells. Our data suggest potential approaches to correct lymphocyte reconstitution defects in RA patients receiving lymphocytotoxic therapies and provide further insights into the disease process itself.

## Abbreviations

ACR = American College of Rheumatology; CRP = C-reactive protein; ELISA = enzyme-linked immunosorbent assay; IL = interleukin; OA = osteoarthritis; PBMC = peripheral blood mononuclear cell; RA = rheumatoid arthritis; TNF = tumour necrosis factor; TREC = T-cell receptor excision circle.

## Competing interests

The author(s) delcare that they have no competing interests.

## Authors' contributions

Frederique Ponchel designed, optimized and performed the work on TREC quantification, T-cell differentiation, ELISA and statistics. Robert J. Verburg provided clinical sample (RA, Leiden). Sarah J Bingham provided clinical sample (RA, Leeds). Andrew K Brown provided clinical sample (RA in remission, Leeds). John Moore provided clinical sample (RA, Sydney). Andrew Protheroe provided clinical sample (cancer, Leeds). Kath Short collected clinical samples (cancer, Leeds). Catherine A Lawson processed clinical samples. Ann W Morgan provided clinical sample (RA, Leeds). Mark Quinn provided clinical sample (RA, Leeds). Maya Buch provided clinical sample (RA, Leeds). Sarah L Field provided technical support. Sarah L Maltby provided technical support. Aurelie Masurel provided technical support. Susan H Douglas provided technical support. Liz Straszynski provided technical support. Ursula Fearon provided technical support. Douglas J Veale provided provided support. Poulam Patel is Head of Department (cancer, Leeds). Dennis McGonagle provided support (Leeds). John Snowden provided support (Sheffield). Alexander F Markham is Head of Department (Leeds). David Ma is Head of Department (Sydney). Jacob M van Laar provided support and clinical material (Leiden). Helen A Papadaki is Head of Department and provided clinical samples (Heraklion). Paul Emery is Head of Department (Leeds). John D Isaacs is Head of Department (Leeds).

## Figures and Tables

**Table 1:** Rheumatoid arthritis patients with active or stable, well controlled disease and control individuals

Parameter	Controls	Active RA	OA	RA in remission
*n*	34	28	12	36
Age (mean ± standard deviation [range]; years)	48 ± 16 (24–62)	51 ± 17 (20–83)	60 ± 9 (49–73)	48 ± 11 (25–67)
Sex (male/female)	6/17	9/28	3/9	7/29
Disease duration (mean ± standard deviation [range]; years)	NA	5.1 ± 7.5 (0.1–37)	NA	9.3 ± 6.8 (2–28)
Remission duration (mean ± standard error [range]; months)	NA	NA	NA	29 ± 29 (6–144)
CRP (mean ± standard deviation [range]; mg/l), below /above detection^a^	NA	55 ± 52 (5–164), 0/28	NA	3.5 ± 5.2 (0–12), 23/13

^a^C-reactive protein (CRP) values <5 mg/l are considered below the detection range. CRP values <10 mg/l are considered normal among the local population. NA, not applicable; OA, osteoarthritis; RA, rheumatoid arthritis.

**Table 2:** Patients receiving depleting therapies

Patients	*n*	Age^a ^range (median; years)	Sex (male/female)	Graft manipulation
RA cohort 1	9	32–61 (42)	2/7	Unmanipulated
RA cohort 2	16	43–55 (47)	4/12	CD34 selection
RA cohort 3	6	24–61 (39)	3/3	CD34 selection, T-cell depletion
Solid tumours	7	39–64 (44)	2/5	Unmanipulated
Lymphoma/sarcoma	7	22–64 (46)	5/2	Unmanipulated
Systemic vasculitis (depleting antibody therapy)	3	43–61 (52)	2/1	Not applicable

^a^Age at time of transplantation. RA, rheumatoid arthritis.

**Table 3:** Patients in clinical remission satisfying or not satisfying the American College of Rheumatology criteria for remission

Parameter	Remission by ACR criteria^a^	Nonremission by ACR criteria^a^
*N*	17	19
Age (mean ± standard deviation [range]; years)	48 ± 11 (28–67)	54 ± 11 (39–67)
Sex (male/female)	3/14	4/15
Disease duration (mean ± standard deviation [range]; years)	9.8 ± 6.6 (3–25)	9.7 ± 6.3 (2–28)
Remission duration (mean ± standard deviation [range]; months)	26 ± 16 (6–60)	30 ± 36 (6–144)
CRP (mean ± standard deviation [range]; mg/l), below/above detection^b^	3.0 ± 3.8 (0–10), 10/7	4.0 ± 5.2 (0–12), 13/6

^a^American College of Rheumatology (ACR) remission criteria : less than 15 min morning stiffness; no fatigue; no joint pain; no joint tenderness or pain on motion; no swelling of soft tissue in joint or tendon sheaths; and <30 mm/h erythrocyte sedimentation rate. ^b^C-reactive protein (CRP) values <5 mg/l are considered below the detection range. CRP values <10 mg/l are considered normal among the local population.
